# Health and Well-Being of Persons of Working Age up to Seven Years after Severe Traumatic Brain Injury in Northern Sweden: A Mixed Method Study

**DOI:** 10.3390/jcm11051306

**Published:** 2022-02-27

**Authors:** Maud Stenberg, Britt-Marie Stålnacke, Britt-Inger Saveman

**Affiliations:** 1 Department of Community Medicine and Rehabilitation, Rehabilitation Medicine, Umeå University, 901 87 Umeå, Sweden; britt-marie.stalnacke@umu.se; 2 Department of Nursing, Umeå University, 901 87 Umeå, Sweden; britt-inger.saveman@umu.se; 3 Centre for Research and Development in Disaster Medicine, Department of Surgical and Perioperative Sciences, Section of Surgery, Umeå University, 901 87 Umeå, Sweden

**Keywords:** severe traumatic brain injury, well-being, health, long-term perspective, mixed method

## Abstract

Purpose: To explore the health and well-being of persons seven years after severe traumatic brain injury (STBI). Material and methods: Follow-up of 21 persons 1 and 7 years after STBI using surveys for functional outcome, anxiety/depression, health and mental fatigue. Interviews were conducted and analysed using qualitative content analysis. Convergent parallel mixed method then merged and analysed the results into an overall interpretation. Results: Good recovery, high functional outcome and overall good health were relatively unchanged between 1 and 7 years. Well-being was a result of adaptation to a recovered or changed life situation. Persons with good recovery had moved on in life. Persons with moderate disability self-estimated their health as good recovery but reported poorer well-being. For persons with severe disability, adaptation was an ongoing process and health and well-being were low. Only a few persons reported anxiety and depression. They had poorer health but nevertheless reported well-being. Persons with moderate and severe mental fatigue had low functional outcomes and overall health and none of them reported well-being. Conclusions: The life of a person who has suffered STBI is still affected to a lesser or greater degree several years after injury due to acceptance of a recovered or changed life situation. Further studies are needed on how health and well-being can be improved after STBI in the long-term perspective.

## 1. Introduction

Traumatic brain injury (TBI) is a leading cause of injury-related disabilities and mortality [1,2,3,4]. Surviving severe traumatic brain injury (STBI) often causes suffering and limitations in daily life, especially among young adults [1,2], and for some, there is a comprehensive and lifelong impact on health and well-being. Injury severity of TBI is defined according to the Glasgow Coma Score (GCS) [5]. Functional outcome following TBI and STBI is assessed using the Glasgow Outcome Scale-Extended (GOSE) [6,7]. GOSE includes functional, physical, emotional and social domains but does not measure fully how these impairments and disabilities affect health and well-being. The definition of health established by WHO in 1948 [8] has been developed with a focus on well-being and the ability to adapt and self-manage one’s life [9]. Health is also described in a contemporary way as disease and disabilities co-existing together with health along a continuum from total health to total absence of health and from something temporary or limited to something more permanent [10]. Well-being covers a more individual, subjective and holistic view and is often described in narratives and interviews on the basis of, for example, personal feelings [11,12]. When the concept Health Related Quality of Life (HRQoL) is used by clinicians and researchers to define long-term satisfaction by the patient-reported outcome, it refers to how specific diseases or treatments affect life but also how they affect health after trauma [13]. Persons with STBI report lower HRQoL compared with the general population [14]. The two concepts of health and well-being are sometimes intertwined and used interchangeably. In accordance with previous research, we suggest in this paper, that health can be measured through self-reporting and described by others [15,16], while well-being is the person’s own interpretation of their health, which can be described in narratives [17]. TBI is one of the most common reasons for physical, emotional and cognitive disabilities [18]. It has an impact on functioning and reintegration into society [19,20,21] and is linked to health and well-being [14]. In an earlier study on individuals with unemployment and disability, persons with TBI reported poor psychological well-being [22]. It is, therefore, of importance to study these areas further in order to help people into employment and to gain a better understanding of psychological well-being after TBI [23]. However, since treatment and outcome may differ between women and men, it is also important to study gender differences [24]. Initially, low GCS is in many cases not equivalent to severe outcomes, as described in other studies [25,26] and is also of interest to be studied further. Many studies have focused on responses by proxy [27], but self-reported health and well-being as described by the injured person are of importance. This can be accomplished through self-reported measures and interviews despite STBI [28]. Outcomes after STBI differ three months after STBI, and the outcomes can range from fully recovered to death [29]. However, severe cognitive impairment and impaired self-awareness are STBI consequences [30,31] that are commonly associated with disability and reduced health and well-being [32,33]. High energy trauma is prioritised in trauma triage because it is known such trauma causes more severe injury, but it is also important to be aware of low energy falls [34]. Repeated TBI is known as a risk factor for outcome [35]. Most follow-up studies on STBI have used a quantitative design with validated instruments [36], although there have also been qualitative studies [37,38]. The use of both surveys and interviews for a more complete evaluation of the outcome is recommended because validated instruments used by the injured person and proxy have a different input on what a good outcome is. Moreover, the outcome from interviews can be influenced by personal factors or the adjustment that has taken place over time [39]. A mixed-method study covering both health by self-reported surveys and well-being by narrative interviews, which enables an integration of results [40,41], can provide additional health information for trauma and STBI afflicted persons [42,43,44]. In order to find out what a mixed-method can add concerning similarities and differences as well as new insights in the results between surveys and interviews, the aim of this study was to explore an overall perspective of health and well-being for persons who had suffered a severe traumatic brain injury (STBI) seven years previously.

## 2. Material and Methods

### 2.1. Design

A mixed-method was chosen in order to allow the drawing of inferences from both quantitative and qualitative findings in response to the purpose of the study. A convergent parallel mixed method was conducted.

### 2.2. Participants

In an earlier Swedish-Icelandic multi-centre study (the Probrain study) where 5 of 6 university hospitals in Sweden and one in Iceland participated (*n* = 114) [29,45], 37 patients with STBI were recruited prospectively to the Regional Neurotrauma Centre in northern Sweden during 2010–2011, as part of the multi-centre study. Inclusion criteria were age 18–65 years, with acute STBI with the lowest non-sedated GCS 3–8 within 24 h post-trauma. The exclusion criterion was death within 3 weeks after injury. Initial severity in the Probrain study was GCS median 6 (3–8). The Regional Neurotrauma Centre in northern Sweden are responsible for approximately one million inhabitants in an area corresponding to almost half of Sweden with both urban and rural areas. Patients were assessed at 3 weeks, 3 months and 1 year after trauma. Of the 37 injured persons from the north of Sweden, there were 28 survivors at follow-up 7 years after injury. Two persons were not reachable. Two persons declined participation: one of them had a full recovery and the other one gave no reason. Three persons had either answered only a questionnaire or only participated in interviews and were not included. In this study, there were 21 participants, two-thirds of whom were men. For those who participated in this study, GCS was median 6 (3–8). The first author (M.S.), who had been in contact with the injured persons in earlier follow-up studies [45,46], contacted the injured person or their legal trustee and informed them verbally and in writing about the study and obtained their written consent.

### 2.3. Procedure

#### 2.3.1. Mixed Method

In this study, quantitative data from questionnaires were merged with narrative interviews in a convergent parallel mixed method in order to explore aspects of health and well-being for persons who had suffered STBI 7 years earlier. Mixed method utilises the respective strengths of quantitative and qualitative research and allows the comparing or combining of results, the challenging of theoretical assumptions and the development of new theories for a better understanding and to bridge the respective weaknesses of the two methods. Parallel analysis is a widely used design in mixed method [41]. The explanatory sequential design is frequently used in trauma studies [42] but in this study we used the parallel convergent design [40]. There were 3 main methodological phases. Firstly, data collection of 2 parallel types of data on the same topic. The data were then analysed separately, and equal value was used. The results from the 2 datasets were thereafter merged and brought together into an overall interpretation. The merging step included comparing results, how they can relate to each other, i.e., the 2 results were combined to facilitate the interpretation.

#### 2.3.2. Quantitative Data Collection and Analysis

In this study, quantitative data were collected through a questionnaire containing background questions and the following instruments: GOSE for functional outcome [6], HADS [47] for emotional health, EQ-VAS [48] as a self-report of overall perceived health, and MFS for mental fatigue [49]. All instruments had closed-ended questions and were distributed at 1-year and 7-year follow-up except MFS. The questionnaires were sent by mail in conjunction with interviews.

##### Instruments

Glasgow Outcome Scale-Extended, GOSE

GOSE evaluates functional outcome after STBI with regard to 8 categories, with a span from “death” (score 1) to “upper good recovery” (score 8). The categories were independence at home, shopping, work, social activities, leisure activities, family, friendship or other problems after TBI. In this study, we used good recovery (GOSE 7–8), moderate disability (GOSE 5–6) and severe disability (GOSE 3–4). The GOSE has good interrater reliability and validity [50].

Hospital Anxiety and Depression Scale, HADS

HADS is an established screening tool for anxiety and depression and has previously been used for patients with STBI [51]. It consists of 14 items organised as 7 items in 2 subscales, HADS-depression (HADS-D) and HADS-anxiety (HADS-A). Both subscales were assessed on a 4-point Likert scale (range 0–3), with the sum of each subscale as the total score (range 0–21). Cut-offs were 8 or higher for both subscales and indicate mild to severe depression and anxiety. The HADS has acceptable reliability, sensitivity and specificity in various populations [52].

Euro-QoL-Visual Analogue Scale, EQ-VAS

The EQ-VAS [41] measures self-reported overall health on a vertical visual analogue 0–100 scale, where the end points were labelled with 100 denoting the best imaginable health and zero as the worst. The EQ-VAS can be used as a quantitative measure of health outcome and is also used in studies of TBI patients (15,16). The participants were asked to mark their health status on the VAS scale. This instrument was validated and has good reliability [53].

Mental Fatigue Scale, MFS

MFS contains 15 questions about common daily activities with 4 alternatives and were associated with “affective, cognitive and sensory symptoms”. It also includes questions about sleep and daily variation of symptoms. This instrument uses a rating based on “intensity, frequency and duration”; the higher the score, the more severe the symptoms [49]. In this study, we used mild (10.5–14.5), moderate (15–20) and severe (>20) mental fatigue.

##### Statistics

Data were analysed using SPSS, version 25.0 for Windows. Data were reported as frequencies, mean or median. Non-parametric tests were used as the sample was small and/or not normally distributed. Statistical significance was set at *p* < 0.05. Wilcoxon’s sign rank test was used for the study of paired observation variables. The Mann–Whitney U test was used for comparison of continuous variables. The Spearman correlation coefficient was used for the analysis of bivariate correlation.

#### 2.3.3. Qualitative Data Collection and Analysis

A total of 21 narrative and semi-structured audio-taped interviews were performed as a family interview with the injured person together with 1–3 other family members. The interviews were transcribed verbatim. Only the texts from the injured persons were used to achieve the purpose of this study. One of the questions posed to the injured persons was how they perceived their recovery and well-being. Most interviews were carried out in the participant’s home; otherwise at the participant’s workplace or at the researcher’s office. Three interviews were conducted by telephone or by video conference due to the great distances in northern Sweden. Qualitative content analysis was used to explore manifest interview text [54,55]. The text was read through to get a sense of the content. Then meaning units were sorted out and coded to meet the aim of the study. Thereafter, the codes were amalgamated into 2 categories, each with 3 subcategories.

### 2.4. Ethical Considerations

This study was approved by the Ethical Review Board, Umeå, Sweden (No. 2016/444-31). Written informed consent was obtained from the participants who were informed they were free to withdraw from the study at any time. There were no withdrawals. During the interview, some participants became emotional and even cried now and then. When this happened, they were asked if they wanted to take a break or stop the interview. No one wanted to stop the interview. A psychologist consultant was available if anyone needed it. The researchers had considered the additional burden that a mixed method study implies, i.e., that the participants were being asked to do both an interview and a survey and had taken steps to minimise that burden.

## 3. Results

### 3.1. Quantitative Results

**Figure 1:**
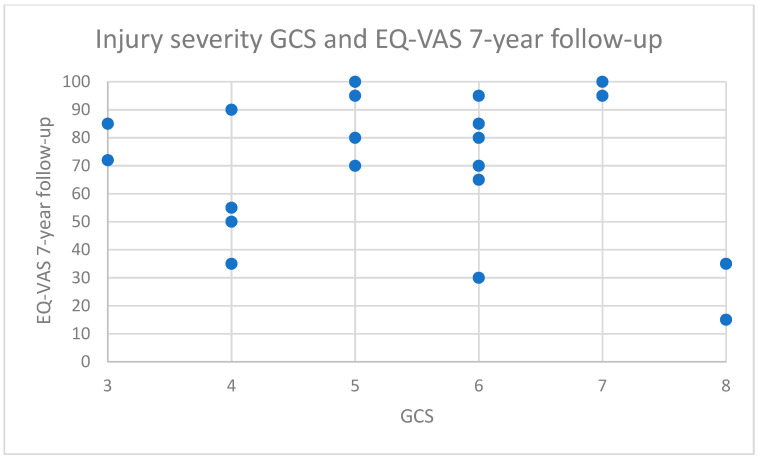
Injury severity on GCS at time of admission and EQ-VAS (overall health) at 7-year follow-up.

For demographic characteristics, see Table 1. For functional outcome measured by GOSE, self-reported health by EQ-VAS, self-reported anxiety and depression by HADS measured at 1-year and 7-year follow-up, and mental fatigue MFS at 7-year follow-up, see Table 2. For injury severity in relation to functional outcome, see Figure 1.

#### 3.1.1. Injury Severity, High Energy Trauma and Previous Brain Injury That Required Hospitalisation Compared with Functional Outcome and Overall Health at 1-Year and 7-Year Follow-Up

There was no significant correlation between initial injury severity and overall health at 1-year and 7-year follow-up (Table 3). Six of the seven women and two of the fourteen men had suffered high-energy trauma. There was no significant difference between persons injured by high energy trauma and persons with no high energy trauma regarding health or functional outcome at 1-year and 7-year follow-up (Table 4). In addition to STBI, one person had an extra-cranial injury (incomplete thoracic spinal cord injury) with overall health rated low at both 1-year (EQ-VAS 10) and 7-year (EQ-VAS 15) follow-up. Seven persons with a previous brain injury that required inpatient care scored significantly lower health at 1-year follow-up (*p* = 0.038) and at 7-year follow-up (*p* = 0.002) compared with those without earlier brain injury that required inpatient care (Table 4).

#### 3.1.2. Functional Outcome and Overall Health at 1-Year and 7-Year Follow-Up

Health on EQ5D-VAS and functional outcome on GOSE for all the participants were rated high both 1 year after trauma and after 7 years (Table 2). Functional outcome 1 year after trauma was median 7 (GOSE 3–8), i.e., “lower good recovery with minor physical or mental deficit” and unchanged at 7-year follow-up (Table 2). A significant difference was found between women and men regarding functional outcome: women had lower scores both at 1-year (*p* = 0.046) and 7-year (*p* = 0.046) follow-up. There was a significant positive correlation between functional outcome and overall health at 1-year (r = 0.513 *p* = 0.017) and 7-year follow-up (r = 0.614 *p* = 0.003), which indicates that higher functional outcome is related to better overall health (Table 3). Participants that scored severe disability (GOSE 3–4) scored significantly lower for health (*p* = 0.013) compared with persons with moderate and good recovery (GOSE 5–8). There was no significant difference in health scores for persons with moderate disability (GOSE 5–6) compared with persons with good recovery (GOSE 7–8) (*p* = 0.078).

#### 3.1.3. Changed Livelihood, Post-Traumatic Epilepsy, Mental Fatigue, Functional Outcome and Overall Health at 1-Year and 7-Year Follow-Up

Changed livelihood, such as ongoing vocational rehabilitation, adapted work or sick leave because of STBI, was relevant for five men and five women at 7-year follow-up. These persons scored a significant deterioration in health (*p* = 0.003) compared with persons with unchanged livelihood (Table 4). Eight persons had medication for post-traumatic epilepsy at 7-year follow-up (Table 1). They scored significantly lower on functional outcome, GOSE at 1-year (*p* = 0.046) and 7-year follow-up (*p* = 0.016) compared with the other participants with STBI (Table 4). Mental fatigue was reported by 40% of the participants. Persons with moderate and severe mental fatigue at 7-year follow-up had a low functional outcome (GOSE 3–5). A significant negative correlation (r = −0.843, *p* < 0.01) at 7-year follow-up was found between mental fatigue and health, indicating that higher mental fatigue was related to poor health (Table 3).

#### 3.1.4. Anxiety, Depression and Overall Health at 1-Year and 7-Year Follow-Up

A majority of the participants (17/21) scored <8 on HAD, indicating no anxiety or depression at both follow-ups. There was a significant negative correlation between self-reported overall health and anxiety and depression both at 1-year follow-up—anxiety (r = −0.539, *p* = 0.017), and depression (r = −0.466, *p* = 0.044), and at 7-year follow-up–anxiety (r = −0.500, *p* = 0.025), and depression (r = −0.790, *p* < 0.001). Thus, no anxiety or depression indicates high perceived health.

### 3.2. Qualitative Results

The qualitative results were presented as two categories, each with three subcategories. The first category was characterised by adaptation for well-being, and the second by the transformation process to well-being (Table 5).

#### 3.2.1. Adaptation for Well-Being

Adaptation for well-being based on personal feelings is characterised by having the ability to adapt, being able to address the difficulties of adaptation, and setting reasonable goals.

##### Having the Ability to Adapt

Having a variety of strategies such as a positive outlook on life, being able to use past experiences, having the ability to use compensatory strategies, having a pragmatic lifestyle, going ahead and having a family, was described as being helpful for adaptation and well-being. There were small things that made daily life difficult, but they were accepted. Participants described being aware that life goes up and down and bearing in mind that a damaged brain finds new ways, and they also referred to themselves as being lucky. Participants described disabilities such as balance problems, hearing loss, becoming blind in one eye, mental fatigue, post-traumatic epilepsy, irritability, pain and impaired memory even though they reported good recovery. Frequently, this only came up in passing at the end of the interviews or in response to a direct question, even though they had described their recovery as being good.

*“The injuries I have are permanent and it will probably be like this for ever”……“I’m learning to live with it,”……“I’m the sort of person who chooses to be happy about what is good instead of being sad about what is not good so I am always positive”……“We enjoy the place where we live and do things that we usually do so we say, enjoy your life, this is quality of life. So in the future, we will continue to feel good and take care of each other. You can always find solutions to things. I don’t have much to complain about—I can walk, I can eat, I can dress myself, so what’s the problem? So compared with many others”……“So it’s just a matter of choosing how to act”……“I do not attempt more difficult things than I can manage quite easily.”*

(Female, 36 years)

##### The Difficulties of Adaptation

The participants described difficulties in understanding the extent of their STBI as being something life-threatening. They had no real experience of what had happened due to the initial weeks of amnesia and neuro-intensive care. Comparing how life was before STBI and how it had become afterwards impeded adaptation and well-being. Adaptation was a continuous, ongoing process for those with severe impairments. Existential changes were described, how their life had been turned upside down, how a new perspective had led to new decisions and compelling reasons to change lifestyle due to STBI with long-term disabilities and new experiences. Denying disabilities, feelings of guilt, shame, loneliness, isolation and avoidant behaviour were described as being obstacles which they had to overcome and which had affected their adaptation and well-being.

*“Yes. Umm, yes, I’m getting more and more depressed, I have become more depressed since this happened because it is always at the back of my mind, my head ruined so much for me. Otherwise I haven’t noticed any other changes after my injury. I guess my memory can be a bit poor sometimes. So these days, I generally have a feeling of sadness. I have almost lost the joy of living, my life feels like that today.”*

(Male, 27 years)

##### Reasonable Goals as a Way to Adapt

The participants described an ability to find new paths for themselves or for others. The ability to adapt is dependent on reasonable goals being set and that they are feasible. For most of the participants, their overall goal was to achieve independence and meaningful everyday life. Taking care of the family, achieving good home conditions, having meaningful leisure time in accordance with the new conditions, and economic independence were all important factors. Because they had been through life-threatening trauma, another successful way to move forward was to avoid drugs, alcohol and a new head injury, to invest in security and in that way reach their set goals. Participants with major disabilities described how their social situation, including their family, social network, social care interventions, housing and rehabilitation, was a prerequisite for independence and suitable goals to be achieved for well-being.

*“Yes, I was so much younger when this happened; it’s completely different today. I’m not so keen on alcohol and partying and stuff like that. I’m completely different. I’m much more mature today with a family and my work……… So my future is to take care of the kids (laughs) and take care of my own little family.”*

(Male, 31 years)

#### 3.2.2. The Transformation Process to Well-Being

The participants described a transformation process with new challenges over the years: becoming aware of their disability, finding new ways in life, learning to live with a disability and then achieving well-being.

##### New Challenges

The participants described that returning to ordinary everyday life and achieving well-being had been a challenge over the years. Some participants said this had taken a short time, while others said it is a lifelong task, throughout the process of transformation with disabilities. Some of them described that they had left or tried to leave the incident behind them because they wanted life to move forward and STBI had been emotionally stressful. Remaining disabilities were described as a challenge and were mentioned even though some of them had described themselves initially as being completely restored. Role changes in families and in working life, difficulties in leisure time, and maintaining social relationships were described as new challenges that affected well-being. Participants with continuing severe disabilities described how daily life in itself was a challenge, how they were dependent on others, and, in many cases, did not have a meaningful everyday life.

*“But just remembering things! That’s the worst! I figure it out in the end. I usually do and when I have to tell someone something, it may take several hours for me to figure things out, but I do.”*

(Female, 55 years)

##### Awareness of Disability

The participants described they began the process of acceptance when they became aware of their disability instead of ignoring or not noticing it. After becoming aware, the participants described their expectations and hopes regarding improvement, a wish to go further in order to achieve well-being. No one had asked for emotional or cognitive rehabilitation, and they described that the rehabilitation they had been given had focused on physiotherapy. Now, 7 years later, it was disappointing that they had to be, as they put it, responsible for their own rehabilitation. In their opinion, more support and rehabilitation were needed in order for them to improve further and achieve well-being.

*“Yes, at the start, during the first year, I assumed I would be able to recover but 7 years later, I have realised that I will not return to the same level where I once was. I know that. I am fully aware of that.”*

(Female, 36 years)

##### Living with a Disability

Living with a disability was described as being able to cope, do things differently, avoid obstacles and overcome challenges. Continuing living with a severe disability was described as frustrating, causing loneliness in everyday life, exclusion from work, social networks, leisure activities and sometimes also from the family. Striving to become more independent and to take personal responsibility was described to make it easier to accept a disability and accept changes in life and achieve well-being.

*“……I can´t talk as I would like to…nothing. Nothing. I’m so damn lonely.…Yes, tears sometimes come when it’s a little hard. Yes, but it’s just a matter of accepting that this is how it is”.*

(Male, 57 years)

### 3.3. Merged Results and Interpretation

The qualitative and quantitative results complement each other since the qualitative results are more explanatory than the quantitative results and cover other aspects. One way in which they differed was that the qualitative results showed an ongoing process during the years following an STBI injury, while the quantitative results showed the impact on health status at certain points in time (after 1 year and 7 years).

Participants described a process of transformation and adaptation, from being a severely injured person with STBI to striving to become an “ordinary” person with a sense of well-being, ending up either with or without a disability in a long-term perspective. Their lives were still impacted to a greater or lesser extent 7 years later by the fact they were a person with STBI. However, a good recovery, high functional outcome and overall good health were relatively unchanged between 1-year and 7-year follow-up. Well-being was described as a recovered or changed life situation that had been accepted. The family was described as being important for transformation, adaptation and well-being. Living with STBI had created opportunities for existential changes and positive outcomes, for example, that over the years, alcohol and drug abuse had ceased. Refraining from comparing their life now with how it was before the injury, having a meaningful everyday life, a positive outlook on life, and the ability to use compensatory strategies, such as making use of previous experiences of challenging events, were helpful for adaptation and well-being. These experiences and the process itself could not have been captured through any of the surveys conducted at a given point in time to measure health or functional outcome.

Changed livelihood because of STBI continued to affect health and well-being after 7 years. For persons with a severe disability, adaptation was a continuous and ongoing process and health and well-being were low. Severe disability implied impairment in all daily activities. Over the years, health and well-being decreased as isolation and loneliness increased.

For persons with moderate disability, workability and social and leisure activities were reduced. Their self-estimated health was on the same level as persons with good recovery, but they described poorer well-being. Hope for progress, being able to cope with an adapted job or find new leisure activities could compensate and improve well-being.

Good recovery was reported for just over half of the participants. High functional outcome, self-reported health and perceived well-being were found even though some of the persons still suffered from disabilities. Disabilities was not described in the interview as being a crucial factor that determined their well-being. They had moved on in life, looking for the future and put the STBI behind them. Even after 7 years, the initial disability that had changed family dynamics was still affecting their well-being and they reported that close relatives pointed out minor personality changes even though they felt completely recovered. Participants who now had a new partner in their life described how the new partner was not able to compare things with how they had been before and that was a relief.

Having an STBI caused by high energy trauma was not related to functional outcome or overall health but for the majority of those affected (6/8), low well-being was reported because it affected their ability to work, their social life and leisure time negatively. Women (6/7) were affected by high energy trauma and scored lower on functional outcome and perceived poorer well-being than men but there was no difference between women and men concerning overall health. Despite some difficulties, there were women who had become a mother but needed the support of loved ones to make daily life with children work. A lack of independence and not being able to work were obstacles that affected well-being negatively.

An earlier brain injury that had required hospitalisation was related to a low degree of overall health and perception of well-being. For persons with post-traumatic epilepsy, the functional outcome was also significantly lower compared with those who did not suffer from epilepsy. Post-traumatic epilepsy also affected well-being but not reported overall health. A few persons reported anxiety and depression and scored lower health, but nevertheless, they had a sense of well-being. Persons with moderate and severe mental fatigue had a low functional outcome and low overall health and none of them perceived well-being or were back in work.

STBI still affects the person’s life to a greater or lesser extent several years after injury. Good recovery and overall good health are reported and also better well-being from interviews due to acceptance of a recovered or changed life situation.

## 4. Discussion

By using a mixed method, we were able to examine an overall perspective of health and well-being for persons who had suffered STBI up to seven years earlier. We found that STBI was still affecting their lives to a greater or lesser extent even though good functional outcomes and overall good health were reported. However, participants reported improved well-being due to recovery or acceptance of their changed situation.

The merged results show that a meaningful everyday life, a positive outlook on life, an ability to use compensatory strategies, such as previous experiences of challenging events, were all helpful for adaptation and well-being. These aspects were not assessed by any of the instruments used to measure health or functional outcome. The injured persons described that focusing on overall health and well-being instead of their disability, even with a remaining impairment, was one way of adapting to the new situation. The present study also found that a pragmatic lifestyle, keeping up with the family and having a meaningful everyday life were ways to overcome challenges in the transformation process for adaptation and well-being after STBI. Achieving health and well-being is connected to what each person considers to be acceptable as a normal everyday life and over the years, new perspectives have emerged.

In this study, several participants mentioned an ongoing disability even though they reported good recovery, health and well-being. This supports that it is not the absence of disease or disability that defines health [10] and that long-term follow-up of unidentified disabilities can be valuable. This was also described in an earlier study when full recovery was reported by persons with TBI after 10 years or more, even though they experienced persistent problems that affected their daily lives [56].

The only exclusion criterion at baseline was death within three weeks. One established weakness of many functional outcome instruments and health scales used in TBI studies is that if there is a pre-existing problem, such as a previous brain injury, drug or alcohol abuse, or psychiatric disorder, it is not possible to take it into account or whether there is a need for guidance when completing the questionnaire [57]. As our merged results take both self-reported health and perceived well-being into account, it is possible to see that it does not only reflect the results but also how pre-existing problems affect the situation.

The merged results show that for the participants with a severe disability, i.e., impairment in all daily activities, adaptation was a continuous, ongoing process and health and well-being were low. Over the years, health and well-being decreased as isolation and loneliness increased. Friends withdrew and sometimes their family members too, even if the family was reported to be helpful for adaptation and well-being. In other studies, STBI persons with more severe residual conditions described difficulties adapting to the new situation and highlighted the importance of having a family, close relationships and access to service and rehabilitation [58] and that feeling self-worth and maintaining self-confidence are important for well-being [59]. Severe disability is known to be related to dissatisfaction with health [28]. In a recent study of persons with STBI, it was shown how rehabilitation could help to adapt friendships and support earlier relationships for the injured person several years after the injury [60].

Participants with moderate disability had the same level of health scores as participants with good recovery but described poorer well-being because of reduced work abilities and social and leisure activities. Changed livelihood affected the health and well-being of almost half of the participants in this study. The categorisation of persons with TBI as either working or unemployed does not provide a complete picture since many people who work fewer hours than they did before the TBI are less satisfied and fail to sustain work [61]. Losing their economic independence was an obstacle and had a negative impact on well-being, especially for the women in this study. The participants in this study were in their most productive years; many of them were on sick leave because of STBI and some of them still had ongoing vocational rehabilitation. There is an obvious need for an intervention programme that is adapted to working life [14].

In this study, participants with known alcohol and drug abuse before injury had ceased all such abuse after 7 years, but none of these persons described any meaningful daily activity, thus service and support were still needed for health and well-being. In our study, the women were 10 years younger than the men. For several of them, their STBI had been caused by high energy trauma and they had lower functional outcomes than men and poorer well-being but acceptable overall health. With the exception of the age factor, our study is in line with a recent multi-centre study which found that women reported more severe outcomes, depending on TBI severity and older age [24]. A recent prediction study reported that women, persons who were unemployed before injury and persons with more severe TBI at 10-year follow-up had lower HRQL, suggesting that these persons should perhaps be targeted for regular follow-up [62]. However, there were only seven female participants in our study, and, therefore, it is not possible to draw any major conclusions regarding whether being a woman has any significance for the outcome.

The merged results showed that there was hope for progress for persons with moderate disability, to cope with work or to find new activities that could be a form of compensation and improve their well-being. Low occupational activity 10 years after TBI gives a low rating for psychosocial function [22], and in our study, we found the same for well-being. Both low life satisfaction and poorer well-being highlight the need for interventions that will promote a meaningfully productive life after TBI [23]. It was found that for up to 15 years post-injury, TBI patients experienced poorer general health, social isolation, and fewer opportunities to work compared with matched persons [63]. This is further confirmation that long-term follow-up after TBI should be considered.

Some of the participants with good recovery mentioned in passing some remaining disabilities. They had moved on in life, put the STBI behind them, reported a sense of well-being and were looking to the future. Negative and stigmatizing reactions from the environment because of earlier STBI were described in another study [64].

Persons with previous brain injury and those with post-traumatic epilepsy were affected with regard to both health and well-being and a poorer functional outcome for those with post-traumatic epilepsy. In an earlier study of persons with STBI, at 10-year follow-up, the frequency of epilepsy was nearly the same as in this study, but their scores for depression were six times higher [14]. In our study, only a few participants reported anxiety and depression at both follow-ups. However, the few who reported anxiety and depression scored lower health but nevertheless perceived well-being. In another follow-up study of survivors, 5–7 years after TBI, anxiety, depression and low self-esteem had stronger associations with persistent and new disabilities than initial severity or cognitive impairment [65]. It is, therefore, relevant to investigate these problems again a long time after the injury [65]. Persons with high scores on the mental fatigue scale had low functional outcomes and low overall health. None of them were back in work, which is in accordance with previous research that showed that higher mental fatigue was linked to low workability and employment [66]. These findings were of importance to consider and highlight the need for follow-up for persons even a long time after STBI since they could benefit from treatment and rehabilitation, including interventions for fatigue.

### Strengths and Limitations

A mixed method study enables new insights to be gained concerning a heterogeneous group such as persons with STBI. The lack of depth of the information that can be gained through surveys can be compensated for through interviews. Our results show how useful it is to combine qualitative and quantitative methods whereby one gains another level of understanding where, metaphorically speaking, “one plus one become three”. It is important to gain this “insider” perspective, using qualitative data and being able to understand “what it is like,” realizing that both vulnerability and well-being can co-exist after STBI, thereby gaining a wider perspective [67]. In an earlier study, it was reported that the evaluation of the outcome of rehabilitation requires both subjective and objective outcome measures. In our study, well-being as a subjective outcome can be described due to adaptation to the new situation and personal factors [39].

The strengths of this study are that it was a prospective cohort study comprising a near-total regional cohort population over a period of two years of persons of working age with STBI admitted to a neuro-trauma centre. The first author (M.S.) investigated all the registered cases at 1-year and 7-year follow-up and ensured that data were precisely and completely documented, minimising the amount of missing data and ensuring it was possible for most persons to be included. Exclusion at baseline was persons who did not survive after 3 weeks. The interviews were conducted by the last author (B.-I.S.) together with the first author (M.S.), mostly in the homes of the injured persons, which represented a safe and well-known environment. The first author was well-known to the participants, which helped to make the interview situation comfortable. There was a pre-understanding among the authors, i.e., medical knowledge of STBI rehabilitation (M.S., B.-M.S.), but the last author (B.-I.S.) was unaware of the illness history of each person, thereby limiting the risk for bias.

One limitation of the study was that it was not possible to follow up on all survivors. This study included patients from a near-total regional cohort population (*n* = 37) of STBI 2010–2011 from the earlier Probrain study, and at follow-up, there were 28 survivors. A total of 21 of these answered questionnaires, which was a rather small number for statistical analyses. However, they all participated in interviews, which was then assessed as a relatively large number. With the mixed method approach using both quantitative and qualitative, the number of participants was considered sufficient. Since the age of the participants may reflect the inclusion criteria of persons of working–age, this could probably explain the results of a good recovery. However, the heterogeneity of the age (27–70 years old) of the participants made it difficult to draw any conclusions on differences due to specific age groups.

## 5. Conclusions

The life of a person who has suffered STBI is still affected to a lesser or greater degree several years after injury due to acceptance of a recovered or changed life situation. Further studies are needed on how the health and well-being of all persons with STBI can be improved from a long-term perspective.

## Figures and Tables

**Table 1:** Demographic characteristics at the time of admission and at 7-year follow-up.

	Time of Admission (*n* = 21)	7-Year Follow-Up (*n* = 21)
*Gender, n (%)*		
Men		14 (67)
Women		7 (33)
*Age, median (min-max)*		51 (27–70)
*Age, mean SD*		46 (13)
Men		48 (14)
Women		43 (12)
*GCS, median (min-max)*	6 (3–8)	
Men	6 (3–8)	
Women	5 (3–8)	
*Cause of injury, High energy trauma, n (%)*		
Men	2 (14)	
Women	6 (86)	
*Intensive care (days), median (min-max)*	14 (3–39)	
Men	13.5 (3–39)	
Women	16 (7–23)	
*Inpatient rehabilitation, (days), median (min-max)*	34 (0–117)	
Men	20 (0–117)	
Women	43 (34–89)	
*Livelihoods, employment*		
Unchanged disability pension or social insurance		2 (9.5)
Unchanged retirement pension		1 (5)
Unchanged full-time or part-time work		8 (38)
Ongoing vocational rehab or adapted work		2 (9.5)
Sick leave because of STBI		8 (38)
Other characteristics		
Known alcohol or drug abuse, *n* (%)	5 (24)	0 (0)
Previous brain injury, hospital stay, *n* (%)	7 (33)	
Post-traumatic epilepsy, *n* (%)	1 (5)	8 (38)
Medication for depression, *n* (%)		1 (5)
Co-existing spinal cord injury, *n* (%)		1 (5)

**Table 2:** HADS-A, HADS-D, EQ-VAS score, GOSE, MFS at 1-year and 7-year follow-up.

		1–Year Follow–Up (*n* = 21)	7–Year Follow–Up (*n* = 21)	*p* Value
*HADS–A, median (min–max)*		*3 (0–11)*	*3 (0–13)*	*0.529*
<8 *n* (%)		17 (81)	17 (81)	
≥8 *n* (%)		2 (9.5)	3 (14)	
Missing		2 (9.5)	1 (5)	
*HADS–D, median (min–max)*		*2 (0–12)*	*3 (0–11)*	*0.391*
<8 *n* (%)		18 (85.5)	17 (81)	
≥8 *n* (%)		1 (5)	3 (14)	
Missing		2 (9.5)	1(5)	
*EQ–VAS, median (min–max)*		*75 (10–100)*	*80 (15–100)*	*0.740*
Men		90 (10–100)	87.5 (15–100)	
Women		65 (45–75)	65 (35–85)	
*GOSE, median (min–max)*		*7 (3–8)*	*7 (3–8)*	*0.429*
Men		8 (3–8)	8 (3–8)	
Women		5 (3–8)	5 (3–8)	
Severe disability	GOSE 3–4, *n* (%)	5 (24)	4 (19)	
Moderate disability	GOSE 5–6, *n* (%)	3 (14)	6 (29)	
Good recovery	GOSE 7–8, *n* (%)	13 (62)	11 (52)	
*MFS, median (min–max)*			*9 (0–29)*	
Men (*n* = 10)			3 (0–29)	
Women (*n* = 5)			17 (9–27)	
No mental fatigue	(<10.5), *n* (%)		9 (60)	
Mild mental fatigue	(10.5–14.5), *n* (%)		0 (0)	
Moderate mental fatigue	(15–20), *n* (%)		4 (27)	
Severe mental fatigue	(>20), *n* (%)		2 (13)	

HADS-A = Hospital Anxiety and Depression Scale—Anxiety, HADS-D = Hospital Anxiety and Depression Scale –Depression, EQ-VAS = Euro-QoL-Visual Analogue Scale, GOSE = Glasgow Outcome Scale-Extended, MFS = Mental Fatigue Scale. Wilcoxon U test sign rank test was used for the study of paired observation variables.

**Table 3:** Correlation between EQ-VAS and GCS, GOSE, HADS-A, HADS-D and MFS at 1-year and 7-year follow-up.

1-Year Follow-Up	*n*	EQ-VAS 1-Year Follow-Up	7-Year Follow-Up	EQ-VAS 7-Year Follow-Up
GCS Time of admission	21	r = 0.048, *p* = 0.836	GCS Time of admission	R = 0.028, *p* = 0.903
GOSE 1-year	21	r = 0.513, *p* = 0.017	GOSE 7-year	r = 0.614, *p* = 0.003
HADS-A 1-year	19	r = −0.539, *p* = 0.017	HADS-A 7-year	r = −0.500, *p* = 0.025
HADS-D 1-year	19	r = −0.466, *p* = 0.044	HADS-D 7-year	r = −0.790, *p* < 0.001
	15		MFS 7-year	r = −0.843, *p* < 0.001

The Spearman correlation coefficient was used for the analysis of bivariate correlation.

**Table 4:** EQ-VAS and GOSE comparison between patients with and without: high energy trauma, previous brain injury with a hospital stay, changed livelihood and post-traumatic epilepsy.

	Yes/No (*n*) (%)	EQ–VAS 1–Year Median *p* Value	EQ–VAS 7–Year Median *p* Value	GOSE 1–Year Median *p* Value	GOSE 7–Year Median *p* Value
High energy trauma	8/13 (38/62)	yes 67.5 (10–90)	yes 67.5 (15–95)	yes 6 (3–8)	yes 5.5 (4–8)
		no 90 (35–100)	no 85 (30–100)	no8 (3–8)	no 8 (3–8)
		*p* = 0.104	*p* = 0.161	*p* = 0.374	*p* = 0.414
Previous TBI, hospital stay	7/14 (33/66)	yes 45 (10–100)	yes 35 (15–85)	yes 7(3–8)	yes 5 (3–8)
		no 82.5 (60–99)	no 87.5 (55–100)	no 7.5 (3–8)	no 8 (4–8)
		*p* = 0.038	*p* = 0.002	*p* = 0.360	*p* = 0.149
Changed livelihood	10/11 (48/52)	yes 67.5 (10–95)	yes 60 (15–95)	yes 5(3–8)	yes 5(3–7)
		no 90 (45–100)	no 90 (50–100)	no 8 (3–8)	no 8 (3–8)
		*p* = 0.061	*p* = 0.003	*p* = 0.005	*p* = 0.020
Post-traumatic epilepsy	8/13 (38/62)	yes 75 (35–95)	yes 72 (30–95)	yes 5 (3–8)	yes 5 (3–8)
		no 75 (10–100)	no 82.5 (15–100)	no 8 (3–8)	no 8 (4–8)
		*p* = 0.743	*p* = 0.360	*p* = 0.046	*p* = 0.016

Non-parametric test independent samples, Mann–Whitney U was used for comparison of continuous variables.

**Table 5:** Perceptions 7 years after STBI.

Categories	Subcategories
Adaptation for well-being	Having the ability to adapt
	The difficulties of adaptation
	Reasonable goals as a way to adapt
The transformation process to well-being	New challenges
	Awareness of disability
	Living with a disability

## Data Availability

The datasets generated and/or analysed in this study are not publicly available as the Ethical Review Board has not approved the public availability of these data.
